# Mechanisms at the Intersection of lncRNA and m6A Biology

**DOI:** 10.3390/ncrna12010004

**Published:** 2026-01-31

**Authors:** Samuel J. Gonzalez, Edgardo Linares, Allison M. Porman Swain, Aaron M. Johnson

**Affiliations:** 1 Department of Biochemistry, Anschutz Medical Campus, Molecular Genetics University of Colorado, Aurora, CO 80045, USA; samuel.gonzalez@cuanschutz.edu (S.J.G.); edgardo.linares@cuanschutz.edu (E.L.); 2 Molecular Biology Program, Anschutz Medical Campus, University of Colorado, Aurora, CO 80045, USA; 3 Department of Chemistry, United States Air Force Academy, Colorado Springs, CO 80840, USA; allison.swain@afacademy.af.edu

**Keywords:** long noncoding RNA, epitranscriptomics, N6-methyladenosine (m6A), gene regulation

## Abstract

This review provides a thorough survey of long noncoding RNAs that bear the RNA modification N6-methyladenosine (m6A) and current work to understand the resulting mechanistic and biological consequences. We give an overview of lncRNA and m6A biology first, describing the writers, erasers, and readers of m6A and their targeting of lncRNAs. Next, we give an in-depth review of the field of nuclear lncRNAs that regulate chromatin and their regulation via m6A. We then describe the growing appreciation of liquid–liquid phase separation properties in lncRNA and m6A biology. Finally, we cover examples of cytoplasmic lncRNAs regulated by m6A. Overall, this review aims to emphasize how epitranscriptomics influences noncoding RNA mechanisms to provide additional layers of regulation, integrated into downstream biological processes.

## 1. Introduction

RNA modifications provide an added level of regulation within RNA biology mechanisms. In many cases, enzymatic addition of chemical groups to RNA has the ability to modify the function or fate of the RNA molecule. These epitranscriptomic regulatory mechanisms are often mediated by proteins that can distinguish the unmodified and modified state. Specific protein domains within RNA modification “reader” proteins have the capacity to bind with higher affinity to modified RNA, with added specificity from the surrounding nucleotide sequence. One of the most common enzymatically catalyzed modifications is N6-methyladenosine (m6A). While m6A occurs on certain positions on rRNA, it is also the most abundant modification of mRNAs, added as they are transcribed. Because mRNAs and long noncoding RNAs (lncRNAs) are both transcribed by RNA Polymerase II (Pol II), lncRNAs are also frequently m6A-modified. LncRNAs, generally defined as Pol II-transcribed RNAs greater than 200 bases with no significant protein-coding potential, have a wide variety of functions in different compartments in the cell. Recent cataloging of human lncRNAs by GENCODE [1] puts the number at ~35,000. Many of these RNAs are directly associated with a protein coding gene as an opposite-direction bidirectional transcription or antisense event. Some annotated lncRNAs derive from enhancer transcription. Therefore, only a fraction are independent genes and a smaller fraction have so far been characterized for a specific, RNA-dependent function. As with protein-coding genes, only a portion are expressed in any one cell type. From our own m6A profiling work, we observe ~500 expressed lncRNAs with at least one m6A site [2], demonstrating a fairly widespread intersection of m6A and lncRNA biology. Modification by m6A can affect a lncRNA in many ways, from altering its stability, regulating its molecular mechanism or subcellular localization, or even regulating the chromatin locus from which the lncRNA was produced. We provide an overview of the intersection of m6A epitranscriptomic regulation and mammalian lncRNA biology in this review. We highlight how this RNA modification can tune the function of noncoding RNA molecules that then can impact the underlying biology or human disease.

## 2. Writers and Erasers of m6A

**Figure 1:**
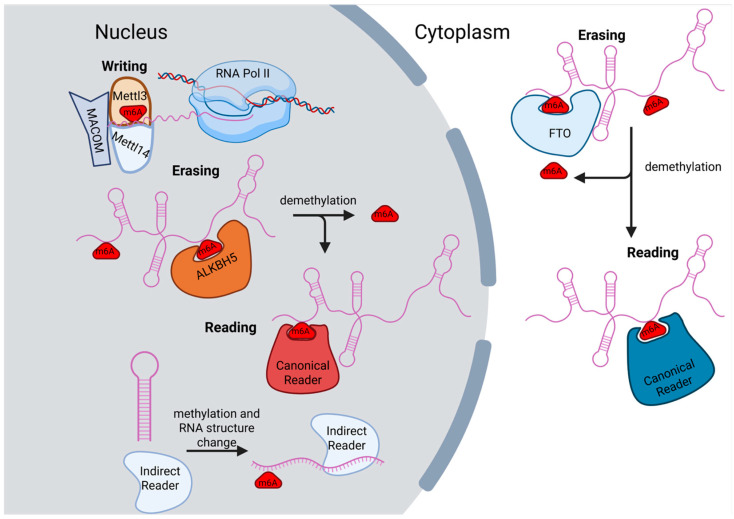
**General m6A machinery.** The primary writer of m6A within mammalian cells is Mettl3/14, in complex with many regulatory subunits (WTAP, VIRMA, AC3H13, HAKAI, and RBM15) that make up the MACOM. Deposition of m6A marks on lncRNAs occurs co-transcriptionally. Readers of m6A can preferentially localize to the nucleus or the cytoplasm, coming in two main types: canonical readers and indirect readers. Canonical readers directly bind m6A, while indirect readers bind the RNA sequence that becomes available due to changes in the secondary structure of the RNA upon m6A methylation. The two primary erasers of m6A are ALKBH5 and FTO; ALKBH5 functions primarily in the nucleus, while FTO functions primarily in the cytoplasm. Created in BioRender. Gonzalez, S. (2025) https://BioRender.com/i0u2r64 (accedd on 21 November 2025).

Modification of RNA with m6A mainly happens as transcription occurs, where nuclear m6A patterns of nascent RNA bear very strong similarity to cytoplasmic patterns [3]. The primary m6A ‘writer’ enzyme complex for Pol II-transcribed RNA consists of a core heterodimer composed of S-adenosylmethionine (SAM)-dependent methyltransferase-like 3 (METTL3) and methyltransferase-like 14 (METTL14) in association with regulatory subunits Wilms Tumor 1-asscoiated protein (WTAP), KIAA1429 (VIRMA), zinc finger CCCH-type containing 13 (ZC3H13), HAKAI, and RNA-biding motif protein 15 (RBM15) (termed m6A METTL-associated complex, MACOM [4]) (Figure 1) [5,6]. This methyltransferase complex targets RNAs containing a ‘DRACH’ consensus sequence (where ‘D’ is any nucleotide but cytosine, ‘R’ is any purine, and ‘H’ is any nucleotide but guanine), with an obligate cytosine downstream of the substrate adenine being essential for methylation [7]. In humans, methyltransferase METTL16 can also generate m6A modifications on mRNA, though these do not happen within the same ‘DRACH’ consensus motif and only a very few substrates are known [8,9]. Methylation by the m6A enzyme complex METTL3/14 is inhibited when the RAC motif is involved in base-pairing [10]. Additionally, recent evidence suggests that the Exon Junction Complex, deposited after splicing, is a negative regulator of m6A deposition within 100–200 nucleotides on either side of splice junctions [11,12,13,14]. This connection helps to explain the enrichment of m6A in longer exons. The regulatory subunits aid in targeting the methyltransferase complex to defined sequences on specific transcripts. For example, for the long noncoding RNA Xist, the RBM15 subunit directs methylation [15].

The removal of m6A marks is carried out by two demethylases, fat mass and obesity-associated (FTO) and AlkB homology H5 (ALKBH5) [16], which are iron (II)- and alpha-ketoglutarate-dependent dioxygenases (Figure 1). The two enzymes have differences in their catalytic mechanisms, with ALKBH5 promoting faster conversion back to adenosine and FTO, producing an N6-hydroxymethyladenosine (hm6A) intermediate [17]. Understanding the specificity between the erasers is an emerging field, with insights on the targeting of specific transcripts, based on perturbation to FTO or ALKBH5 protein levels and following direct changes that occur in the level of methylation at specific m6A sites. Both enzymes have some additional activity reported for other methylated adenosine modifications. FTO can demethylate RNAs, likely in both the nucleus and cytoplasm, based on its localization patterns. A major role for FTO in the cytoplasm is to demethylate N6,2-O-dimethyladenosine (m6Am) 5′ caps [17,18]. ALKBH5 is primarily nuclear, therefore less likely to influence mature RNAs in the cytoplasm. ALKBH5 can be recruited and activated to demethylate mRNA by an adapter protein, RBM33 [19]. Both FTO and ALKBH5 have been shown to demethylate lncRNAs.

## 3. m6A Readers and Their General Mechanisms of Action

Much of the function of an m6A mark is mediated by binding of the mark by proteins called “readers.” Known reader proteins that interact with sites on lncRNAs include YT521-B homology (YTH) domain family proteins (YTHDF1/2/3 and YTHDC1/2), insulin-like growth factor 2 mRNA-binding proteins (IGF2BP1/2/3), Human antigen R (HuR), Leucine-Rich Pentatricopeptide Repeat Containing protein (LRPPRC), and some heterogeneous nuclear ribonucleoproteins (HNRNPC, HNRNPG, and HNRNPA2B1) [20].

Each m6A reader has a unique combination of domains involved in m6A recognition, non-m6A dependent RNA binding, subcellular localization, and protein–protein interactions. These m6A readers can interact with m6A-modified lncRNAs associated with different biological functions, as described below.

### 3.1. Domain Architecture of m6A Readers

m6A readers can be split into two categories: canonical and indirect readers (Figure 1). Canonical readers have m6A recognition domains that directly bind the modified RNA sequence, while indirect readers are indirectly influenced by the m6A mark in their binding to proximal RNA regions. The YTH domain containing proteins and IGF2BPs are canonical readers. The YTH domain is highly functionally and structurally conserved amongst the YTH protein family [21]. The highly conserved residues are involved in an “aromatic cage” consisting of two or three tryptophan residues and another hydrophobic residue surrounding the adenosine. The specificity towards a methylated adenosine is due to π-π interactions between the nucleotide base and the aromatic cage and surrounding cation-π interactions [22]. YTH domain binding affinity significantly decreases for a non-methylated adenosine compared to a methylated one; a similar fold decrease is observed for methylated RNA when one or more of the aromatic cage’s tryptophan residues are mutated. YTH domain family proteins YTHDF1/2/3 have a stronger affinity to m6A due to their low-complexity domains (LCD), forming another hydrophobic alpha helix within the aromatic cage [23]. The YTH domain also has an affinity for the N1-methyladenosine (m1A) modification on RNAs, except in YTHDC2 [24].

IGF2BPs (1/2/3) have two RNA recognition motif (RRM) domains near their N-terminus and four K homology (KH) domains at their C-terminus [25]. Their KH3-KH4 di-domains are essential for m6A recognition, while KH1-2 contributes to their m6A affinity. In silico and molecular analysis showed that the KH4 domain in IGF2BP1 uses a hydrophobic cradle of residues to interact with its m6A target, contributing to higher m6A affinity than IGF2BP2 and IGF2BP3 [26,27]. Through molecular dynamics assays, IGF2BP2 and IGF2BP3 were found to shift the bound m6A from the KH4 domain to binding to the KH3 domain [27].

The HNRNP family is an example of indirect m6A readers that act by recognizing specific sequence motifs that become available due to structural changes caused by m6A modifications on their target transcripts [28]. HNRNPA2B1 has two RRM domains, consisting of two α-helices and four β-sheets, making a sandwich binding pocket [29]. The HNRNPA2B1 conserved RNA binding motif of repeated arginine (R) and glycine (G) residues (RGG box) also contributes to its RNA affinity [30]. HNRNPC and HNRNPG have one RRM domain and acid-rich and glycine-rich regions contributing to their RNA affinity, respectively [31].

Other m6A reader proteins, such as HuR and LRPPRC, have demonstrated affinity for m6A-modified transcripts, but additional biochemical/biophysical studies of their m6A binding domains is still needed [32].

### 3.2. Subcellular Localization

Each m6A reader interacts with its RNA targets in the cytoplasm, nucleus, or both, depending on their distinct subcellular localizations: YTHDF1, YTHDF2, and YTHDF3 are primarily cytoplasmic; YTHDC1 localizes to the nucleus, particularly nuclear speckles; and YTHDC2 is present in both the nucleus and cytoplasm [28]. However, YTH protein localization can change in response to cellular perturbations such as cellular stress. Radiation stress causes YTHDF1 to be phosphorylated and prevents nuclear export, resulting in nuclear accumulation, which then allows YTHDF1 to increase splicing and expression of DNA repair genes [33]. During heat shock stress, cytosolic YTHDF2 moves to the nucleus and binds to m6A sites on Hsp70 mRNA, preventing FTO demethylation and promoting increased translation [34]. The nuclear export protein CRM1 has affinity for all three YTHDF proteins, showing nucleus-to-cytosol potential [35]. O-GlcNAcylation helps mediate YTHDF1 binding to CRM1 and nuclear export; however, whether O-GlcNAcylation plays a role in the other YTHDF proteins’ translocation is unknown [36]. IGF2BPs are mostly cytoplasmic, with their KH domain preventing nuclear accumulation, except IGF2BP3, which can be shuttled to the nucleus [37,38]. HuR is a nuclear-cytoplasmic protein with a shuttling sequence within the hinge region between its RRMs 2 and 3 [26]. LRPPRC is known to be a mitochondrial protein but has been found to associate with RNA in the cytoplasm and nucleus [39]. The HNRNPs are primarily nuclear proteins but can translocate to the cytoplasm [40,41], dependent on specific domains, for example, on HNRNPA2B1 and HNRNPC [40,42].

### 3.3. Consensus Sequence Binding

The m6A consensus sequence, DRACH or RRACH, is commonly recognized by reader proteins, but some readers have specific nucleotide bias or can recognize non-DRACH dependent m6As. Binding and structural analysis showed YTHDC1 has a bias for a guanine at the -1 position from an m6A site (G(m6A)C), with residue stacking and hydrogen bonds with residues around the aromatic cage [43]. The other YTH-containing proteins are known to bind to this motif as well, as shown in CLIP-seq data, but in vitro experiments with different m6A k-mers have shown more flexible binding, with them preferring pyrimidine bases at the −1 and −2 positions and not needing C on the +1 position [44]. YTHDF2 has a lower affinity to m6A sites located on RNA duplexes; this is presumed to generalize to other YTH proteins but has not been formally demonstrated [45]. The KH3-4 di-domains on IGF2BP 1/2/3 have been shown to recognize m6As with the consensus DRACH sequence, including within lncRNAs such as ZFAS1 [46]. These in vitro m6A-transcript experiments show that reader proteins can bind, sometimes better, to m6A within a sequence context that rarely/never exists in the cell, since the motif preference of the methyltransferase dictates which sequences actually become methylated. It is possible that lower affinity interaction of a reader with a methylated RNA is beneficial for allowing downstream steps that would be blocked by binding too tightly.

HNRNPs have an indirect mode of recognizing the m6A consensus sequence. m6A:U base-pairing is weaker than A:U, causing local RNA unfolding which increases RNA accessibility for HNRNPC to the ssRNA. This RRACH–U–tract coupling event where m6A base pairs with poly-U tracts has been dubbed the “m6A switch” model [47,48]. A recent study focused on how HNRNPC interacts with various endogenous m6A-modified RNAs conducted biophysical experiments and computational simulations to refine the model to suggest that m6A causes more subtle conformational changes in RNA structure, not large-scale unfolding, to energetically prime protein binding [49]. m6A switches also contribute to HNRNPG binding to m6A sites flanked by purine-rich regions in mRNAs [50]. HNRNPA2B1 has been shown to bind flanking sequences of the DRACH motif; however, A2B1 binding to short RNAs is directly inhibited by m6A, suggesting a possible m6A switch mode of binding [30]. m6A marks near HuR’s binding motif, AU-rich elements, increased HuR affinity to its site, while m6A marks further from the binding site moderately decreased HuR binding [51]. Whether this is due to m6A switches destabilizing the RNA structure needs to be further explored. LRPPRC has a broad RNA-binding affinity due to its many α-helical structure domains that facilitate RNA binding [52].

### 3.4. m6A Readers That Regulate LncRNAs

Most m6A-methylated lncRNAs can bind one or more of the m6A readers described above. The molecular consequences of reader interaction with lncRNAs is very context-dependent, as is the subsequent integration of the molecular events into a biological and physiological effect. We describe some of these context-dependent m6A reader-lncRNA mechanisms in the following sections of this review. As a more comprehensive catalog of these interactions, we provide, in table form, a list of readers, the lncRNAs they interact with, the molecular outcome, and the physiological/disease context associated with the mechanism (see Supplementary Table S1).

## 4. Role of m6A on LncRNA Structure and Stability

### 4.1. m6A Effect on LncRNA Structure and Accessibility to RNA Binding Proteins (RBPs)

m6A modifications can affect RNA stability in at least two ways, either by modulating the binding of reader proteins that regulate RNA stability or by altering RNA structure which then changes RNA stability. For coding RNAs, m6A can also influence stability by slowing translation.

**Figure 2:**
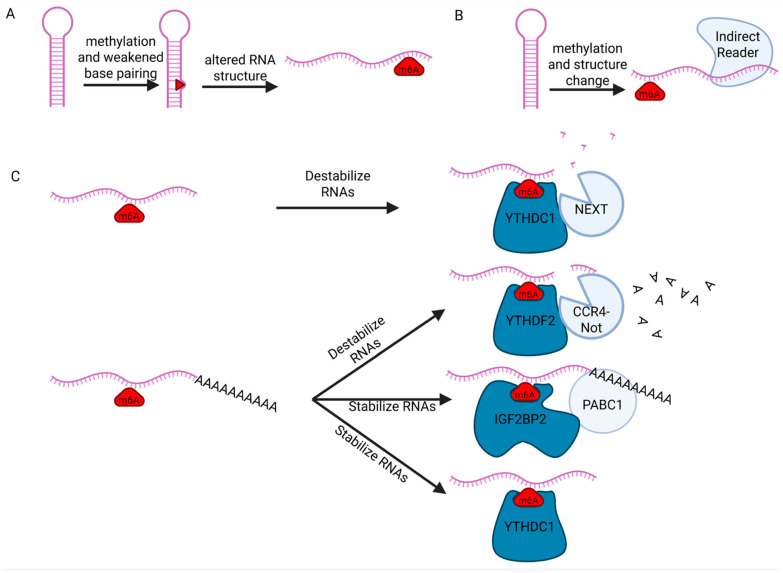
**Effect of m6A on RNA stability and structure.** (**A**) m6A can weaken base pairing, leading to the opening of RNA hairpins or other secondary structure. (**B**) Changes in the structure of RNAs caused by m6A can lead to increased availability of binding sites on the RNA, ultimately increasing the recruitment of indirect readers. (**C**) Depending on which reader is recruited to m6A, the mark can either contribute to destabilization of the RNA (via recruitment of the CCR4-NOT complex, for example), or the mark can contribute to stabilization by recruiting proteins like PABPC1. Created in BioRender. Gonzalez, S. (2025) https://BioRender.com/plaoej4 (accedd on 21 November 2025).

Current evidence suggests that the effect of m6A on the structure of RNAs is sequence and structure context-dependent [45,50,53,54]. For example, m6A on adenosines tend to modestly destabilize their ability to participate in base-pairing (Figure 2A) [45,50,53,54]. For regions of RNA that are single stranded, however, the presence of the m6A mark appears to improve base stacking of the RNA [53]. Taken together, this work suggests that the effect of m6A on RNA structure is likely dependent on the nearby secondary structure of the RNA. Structural changes in RNA driven by m6A are termed the “m6A-switch” (Figure 2C) [47,50,53,54]. These structural changes play a role in the differential recruitment of RBPs to RNAs, based on methylation status [50,54], first highlighted by the effect of m6A on the recruitment of HNRNPC to the MALAT1 A2577 region [47]. In short, HNRNPC normally binds poly-U tracts, one of which is near A2577 of MALAT1 and is normally unavailable due to base pairing with A2577 and its nearby nucleotides. m6A methylation of A2577, however, decreases the stability of the A-U base pairing, leading to a reduction in the stability of the hairpin normally formed and opening this poly-U tract for binding by HNRNPC. The m6A switch also operates for other lncRNAs, where changes in RNA secondary structure due to altered base-pairing caused by m6A methylation drives binding of RBPs [47]. Canonical m6A readers may also help open up binding sites for indirect m6A readers by directly binding the m6A site, thereby increasing the accessibility of the proximal binding site for indirect m6A readers, although more work is required to determine the extent of this mechanism. In short, the field has demonstrated that m6A methylation can help drive structural changes and subsequent stability changes in some instances. Future work in the field will lead to better characterization of how ubiquitous this m6A-switch mechanism is with other lncRNAs and m6A readers in the hopes of identifying some unifying principles surrounding how m6A methylation can intrinsically alter RNA structure and function.

### 4.2. m6A Effect on LncRNA Stability via RBP Recruitment

In tandem with structural changes in RNA caused by m6A methylation, the presence of this mark can also change RNA stability by affecting which RBPs bind to lncRNAs. Whether or not the m6A mark has a stabilizing or destabilizing effect on the lncRNA appears to primarily depend on which m6A reader is recruited to the lncRNA (Figure 2B) [55]. For RNAs in general, YTHDF proteins tend to destabilize the RNAs they bind, while IGF2BPs tend to stabilize them [55,56,57]. The way that YTHDF proteins can destabilize is best showcased by YTHDF2, which can recruit multiple types of RNA metabolism machinery [55,58,59,60]. For example, YTHDF2 directly interacts with the CCR4-Not complex, which deadenylates RNAs to facilitate exosome degradation, reducing the level of the lncRNA PLAC2 [59]. YTHDF2 can also recruit the RNaseP/MRP complex to the lncRNA PLAC2 to degrade the transcript [60]. In both of these cases, YTHDF2 works as a bridge, leveraging the presence of the m6A site on the RNAs to then recruit degradation machinery to these RNAs. IGF2BPs, like IGF2BP2, tend to stabilize m6A RNAs by recruiting RNA stabilizing factors like HuR, MATR3, and PABPC1 [56]. For example, the overexpression of IGF2BP2 in FTL3-ITD acute myeloid leukemia allows for increased stability of the lncRNA DANCR, contributing to increased proliferation and a worse prognosis for patients [61]. Taken together, these examples of YTHDF2 and IGF2BP2 suggest that changes in the stability of RNAs caused by m6A tend to be through the recruitment of an RNA stabilizing or destabilizing protein factor via the m6A reader.

Some readers, however, appear to have different effects on RNA stability, depending on the situation. The m6A reader YTHDC1 is able to stabilize the lncRNA HOTAIR, and loss of YTHDC1 or m6A sites on HOTAIR leads to reduced HOTAIR levels [2]. Furthermore, other work has found that YTHDC1 stabilizes SQSTM1 mRNA and is able to stabilize enhancer RNAs (eRNAs) as well [62,63]. On the other hand, YTHDC1 binding to m6A-methylated PTEN mRNA appears to increase degradation of this transcript [64]. YTHDC1 also increases degradation of m6A-methylated LINE1 RNAs via recruitment of the nuclear exosome targeting (NEXT) complex [65]. Still, in other cases, it appears that YTHDC1 has no effect on the stability of the RNA it binds [66]. Taken together, these data suggest that YTHDC1 may have differing effects on RNA stability depending on the context, likely by differentially recruiting other proteins to affect RNA stability. The major body of work on m6A reader-mediated stability has focused primarily on mRNAs rather than lncRNAs, and therefore these same patterns may not hold true in all cases for lncRNAs. Future work should focus on better understanding how m6A readers alter stability of different lncRNAs, whether through the surrounding RNA sequence, differential ability to recruit effector proteins, or both.

It is important to highlight that robust methods to quantify m6A methylation are needed to ensure careful studies can be performed to evaluate the effect of these marks on stability. While prior work has done well to either make site-specific mutations that prevent m6A methylation and evaluate the effect on the stability and function of these RNAs, or generate knockouts of some of the readers, writers, or erasers of m6A marks and subsequently evaluated how this affected RNAs, more quantitative approaches have been limited thus far [67]. Excitingly, however, new techniques are quickly coming online that hold promise for robust, quantitative evaluation of m6A marks along lncRNAs, allowing for a more careful understanding of the relevance of these m6A mechanisms on lncRNA stability [68,69,70]. While meRIP and associated methods have been a staple for evaluating the amount of m6A methylation, these techniques are not site specific and tend to have difficulty being quantitative. CLIP-based methods have been highly valuable for identifying individual methylation sites, but have lacked a quantitative framework for assessing percentage methylation at each site in a population of RNAs [2,71,72]. New methods, however, like Nanopore sequencing of m6A marks, GLORI, and eTam-seq, hold promise for more quantitative evaluation of m6A methylation [68,69,70,73,74]. These methods for m6A quantification work either through directly sequencing RNA (Nanopore) or indirectly (eTam-seq and GLORI) by converting non-m6A-methylated adenosines into inosines, allowing for them to be read as guanines upon sequencing. These techniques quantitatively measure m6A methylation at specific nucleotides, allowing for more careful evaluation of the magnitude of methylation at an individual site on the transcript’s stability.

## 5. Involvement of m6A in LncRNA-Mediated Chromatin Repression

There are several instances where m6A modification of nuclear lncRNAs supports their ability to promote gene repression and subsequently induce heterochromatin formation. In the following examples, m6A deposition on a lncRNA enables interaction with the nuclear m6A reader YTHDC1 to induce transcriptional repression and in some cases chromatin condensation. This process can occur both in *cis* and in *trans*, and, like most m6A marks, the outcome is context dependent.

### 5.1. Xist

Perhaps the most well-studied example of a lncRNA that induces heterochromatin is X-inactive specific transcript (*Xist*), a ~17–19 kb nuclear localized noncoding RNA. Xist is transcribed from the X chromosome that will undergo X-chromosome inactivation (XCI), a process in which one copy of the entire X chromosome in female mammals is silenced, packaged into heterochromatin [75,76]. *Xist* orchestrates XCI by accumulating over the X chromosome from which it is transcribed and recruiting additional factors that act to silence and condense the chromatin. While the precise mechanism and sequence of events that lead to XCI remains an active area of research, the establishment of silencing involves exclusion of RNA Polymerase II (RNAPII), loss of histone modification associated with transcription, and gain of histone modifications associated with Polycomb repressive complexes PRC1 and PRC2 [77].

The first evidence of potential m6A modification of Xist came in 2015 from multiple independent studies. Mass-spectrometry-based investigations identified two subunits of the m6A methyltransferase complex, WTAP and RBM15, as Xist-interacting proteins [78,79,80]. A pooled shRNA screen identified RBM15 and WTAP as important factors for Xist-RNA-mediated silencing in a transgenic reporter embryonic stem cell (ESC) line [81]. Concurrently, the first single nucleotide m6A mapping in human cells discovered multiple m6A sites in Xist [71].

Following these initial findings, a study from the Jaffrey group found that Xist has multiple binding sites for RBM15 and RBM15B and >60 m6A methylation sites, including a cluster in the A repeat region, that are dependent on RBM15/15B, WTAP, and METTL3 in mouse ESCs. iCLIP revealed that the nuclear m6A reader YTHDC1 bound Xist specifically in regions that were m6A-modified. Experiments in a male mouse ESC line that expresses a doxycycline-inducible Xist on the X chromosome and single-molecule fluorescence in situ hybridization (sm-FISH) to monitor expression of X-linked transcripts demonstrated that this reader was necessary for Xist-mediated silencing (via YTHDC1 knockdown) and sufficient to overcome Xist silencing defects upon knockdown of METTL3 (by tethering YTHDC1 to Xist) [82].

A caveat of these studies is that they were performed in mouse cells that use transgenic Xist expression to establish silencing, with various techniques to measure the level of silencing. Studies in 2019 and 2020 used interspecific XX mouse ESCs to examine endogenous Xist function. To define the contribution of different pathways in XCI, an inducible endogenous Xist and measurement of nascent RNA transcripts (including allelic analysis from the active and inactive X chromosomes) was employed. In this context, with an inducible Xist system, knockout of RBM15 or WTAP showed minimal effects on Xist-mediated transcriptional repression, whereas SPEN and Polycomb were key to the process [83]. In support of this, SPEN (specifically its SPOC domain) was shown to be essential for the initiation of XCI in mouse embryos and mouse ESCs, but dispensable for the maintenance of XCI in neural progenitors [84]. Interestingly, the m6A RNA methylation machinery was found to associate with the SPOC domain of SPEN, suggesting that SPOC may play a role in the m6A methylation of Xist. Using this same interspecific XX mouse ESC system, CRISPR/Cas9-mediated mutagenesis to delete portions of the 5′ Xist m6A region was used to investigate impacts on m6A modification and Xist-mediated silencing. Deletion of the Xist A repeat (where RBM15 binds) abolished m6A modification of the Xist 5′ region and impaired silencing, supporting a role for m6A in this process [85]. A study examining the extensive modular structure of the Xist RNP also supports the function of the essential A-repeat domain in recruiting m6A methylation machinery to Xist, although multiple other proteins are also recruited by this domain, emphasizing that the A-repeat serves as a nucleation center for Xist protein recruitment [86].

To address whether m6A plays a role in Xist stability, a recent 2025 study used interspecific XX mouse ESCs with inducible Xist and a dTAG degron system to rapidly deplete METTL3. An improvement over previous methods to detect transcription from inactive chromosomes, the study also took advantage of chromatin-associated RNA sequencing to detect active transcription from the active and inactive X chromosomes, which could be differentiated due to the interspecific XX chromosomes. METTL3 depletion resulted in rapid loss of m6A transcriptome-wide, including over the Xist m6A peak regions [87]. Interestingly, METTL3/m6A depletion led to an increase in Xist levels, and in turn, in the rate of Xist-mediated silencing, suggesting m6A leads to destabilization of the Xist transcript. Indeed, it was found that m6A-modified Xist transcripts were targets for degradation by the NEXT complex [66]. This adds another layer of complexity to regulation of Xist by m6A, shedding light on its role in Xist stability and dynamics during the process of XCI induction in mESCs.

With most Xist studies performed in mouse cells, it was unclear whether the same m6A function on Xist was present in human cells. To demonstrate the role of m6A on Xist in human cells, a study performed in HEK293T cells, a cell line derived from a human embryonic kidney that has multiple X chromosomes that are silenced but one single active X, found that the knockdown of METTL3 resulted in upregulation of two X chromosome genes, *GPC4* and *ATRX*, as determined by RT-qPCR [88]. Repression was restored by specifically targeting the methyltransferase domain of METTL3 to Xist, suggesting m6A methylation of Xist is key to the repression of X-chromosome-transcribed genes. In contrast, tethering the m6A demethylase FTO to Xist led to a minor but significant upregulation of *GPC4* and *ATRX*, demonstrating that the demethylation of Xist may partially overcome epigenetic silencing of the X chromosome.

**Figure 3:**
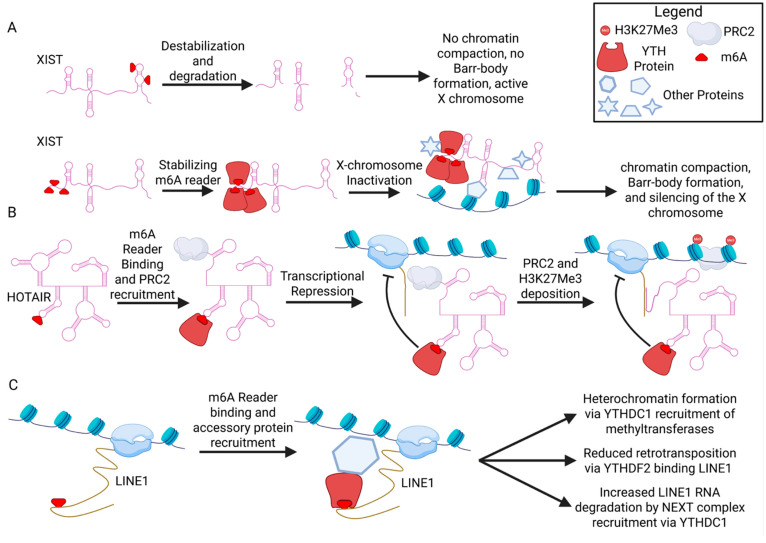
**Role of m6A methylation on lncRNAs in chromatin repression.** (**A**) Xist can undergo m6A methylation near its 3′ end, allowing for the recruitment of the NEXT complex and the subsequent degradation of Xist. Ultimately, this leads to inhibited X-chromosome activation and loss of Barr-body formation (top). Additional sites in the 5′ end of the transcript can also undergo m6A methylation, allowing for the recruitment of YTHDC, contributing to X-chromosome inactivation and Barr-body formation, in conjunction with many other factors (depicted as other shapes). (**B**) HOTAIR undergoes m6A methylation at site A783. This subsequently recruits YTHDC1, which induces initial transcriptional repression of specific loci in the genome caused by HOTAIR. Subsequently, PRC2 is deposited at these loci, leading to long-term repression of these genes. (**C**) LINEs are able to undergo m6A methylation, recruiting different YTH-domain m6A readers such as YTHDC1 or YTHDF2. The binding of these readers recruits other proteins to these LINEs, leading to either increased heterochromatin formation, reduced retrotransposition, or increased degradation of the LINEs, depending on the context. Created in BioRender. Gonzalez, S. (2025) https://BioRender.com/5fix9zi (accedd on 21 November 2025).

Taken together, a multi-pronged model of m6A regulation of Xist has emerged (Figure 3A). A theme in m6A biology is that its function is largely context dependent, with differing functions depending on the RNA transcript and specific sequence that is modified, the cell type the RNA is expressed in, and the location within the cell. The different outcomes observed in these Xist studies likely stem from the different contexts and methods used to induce Xist and to assay silencing. While it is clear that components of the m6A machinery bind to Xist, initiation of XCI and Xist-mediated silencing are likely not absolutely dependent on m6A, although m6A may still play a role in Xist function. There remain a number of unknowns in our knowledge of m6A function on Xist, including the m6A distribution across individual Xist molecules. With new techniques to map m6A in a quantitative fashion, a better understanding of the location and frequency of m6A modification within individual Xist transcripts is possible. How significant a role m6A plays in repression by Xist, and when during development or in different cell types it might have the biggest impact, remain open questions and important areas for future studies to investigate.

### 5.2. HOTAIR

HOX transcript antisense intergenic RNA (HOTAIR) was originally identified in 2007 as a 2.2 kb lncRNA containing six exons that is transcribed from the *HOXC* locus and regulates development by localizing PRC2 and H3 lysine-27 trimethylation (H3K27me3) to the *HOXD* locus in *trans* [89]. HOTAIR has since been extensively studied for its contribution to cancer progression, where overexpression promotes cancer malignancy in many different cancer types including breast, hepatocellular, colorectal, gastric, lung, glioma, cervical, ovarian, and liver cancers [90]. HOTAIR acts as a scaffold to reprogram the chromatin state via its interactions with histone modifiers PRC2 and lysine demethylase 1 (LSD1) to methylate H3K27 and demethylate H3K4 to repress tumor suppressor genes, promoting cancer metastasis [91,92]. The modular structure of HOTAIR folds into four domains to support its scaffolding function, with the 5′ domain 1 interacting with PRC2 and 3′ domain 4 interacting with LSD1 [92,93].

A 2016 proteomic screen of HOTAIR-interacting proteins identified HNRNPA2B1, an RNA binding protein with the potential to be regulated by m6A, as a preferential interactor of HOTAIR [94]. The B1 isoform specifically binds to HOTAIR and target RNAs to enable HOTAIR RNA–RNA interactions, facilitating targeting and repression in *trans* [95,96]. It is also notable that HOTAIR localization to chromatin can repress gene expression in the absence of PRC2, suggesting that the initial transcriptional inhibition by HOTAIR is enabled by other factors [97].

A 2022 study, the first to investigate m6A in HOTAIR, identified multiple sites of m6A modification in HOTAIR, with one specific site at adenosine 783 (A783) being consistently methylated in multiple breast cancer cell lines. Mutation of A783 to uracil (A783U) so that it could no longer be m6A-modified at that site blocked, and in some cases reversed, the cancer-promoting effects of HOTAIR in triple-negative breast cancer cells. This study identified YTHDC1 as a HOTAIR-interacting protein capable of mediating the effects of m6A at A783 (Figure 3B): tethering YTHDC1 to A783U mutant HOTAIR restored its cancer promoting effects, and knockdown of YTHDC1 alleviated transcriptional silencing by HOTAIR [2]. This work suggests that m6A at A783 is required for HOTAIR to stimulate breast cancer progression and could be a promising therapeutic target for cancer patients with HOTAIR overexpression.

Other recent studies have supported a role for m6A modification of HOTAIR. Interactions of HOTAIR with components of the m6A writer complex including WTAP and RBM15, regulation of HOTAIR by methyltransferase member METTL14, as well as regulation of the m6A eraser FTO by HOTAIR have been identified in various cell types and systems [98,99,100]. Overall, this work supports the idea that m6A modification of HOTAIR increases its expression or stability to enable its functions.

A growing body of evidence provides insight into the interaction of m6A modification with HOTAIR function. How specific sites of m6A modification on HOTAIR regulate its structure, interaction with other proteins and RNAs, localization, and impacts on cell fate remains an active area of research. It will be important to determine how distribution of m6A across HOTAIR transcripts influences HOTAIR function in different cell types to gain a better understanding of how mechanisms of m6A modification on HOTAIR drive its cellular impacts.

### 5.3. LINE1

Long Interspersed Nuclear Element-1 (LINE1) is an abundant transposon element which produces RNA that functions as a long non-coding RNA (lncRNA) with crucial roles in gene regulation, genome organization, and development, independently of the retrotransposition activity that is also encoded by the RNA. These nascent LINE1 RNAs are m6A-modified, leading to recruitment of YTHDC1 which facilitates degradation of the LINE1 RNA on chromatin, prior to retrotransposition or nuclear export (Figure 3C) [63]. Without m6A sites on LINE1, its RNAs accumulate, which increases euchromatin formation and LINE1-associated gene expression. YTHDC1 binding to m6A-modified LINE1 is required before SETDB1 deposits H3K9me3 on LINE1 genomic loci, which is essential for mESC identity [101]. Another pathway shows YTHDC1 binding to m6A-modified LINE1 to enable its association with NCL and KAP1, the latter mediating addition of H3K9me3 on the LINE1 loci, which again helps in maintaining early mESC identity [102]. Loss of the eraser FTO increases m6A marks on LINE1 and leads to its lower abundance in mESC [103]. This causes an increase in euchromatin in LINE1-activating genes and dysregulated embryonic development. m6A marks appear to be more abundant on younger LINE1 elements, especially LINE1HS (a.k.a. L1PA1) [104]. m6A marks on LINE1 and subsequent binding of YTHDF2 prevents retrotransposition, which aids in human germline development [105]. This highlights how m6A marks on LINE1 elements act in maintaining gamete and early embryonic development by recruiting m6A readers to destabilize the transposon and lock down their loci. Similarly, m6A marks on endogenous retroviruses act as guardrails for these transposable elements and preserving cellular integrity [106].

## 6. m6A-Modified Enhancer RNAs

eRNAs are RNAs transcribed from enhancer regions (specific DNA sequences that enhance the transcription of associated genes) and range from ~50–2000 nucleotides in length. eRNAs tend to be bidirectionally transcribed by RNA Pol II, 5′ capped, but are not often polyadenylated [107]. eRNA abundance is often correlated with the activity of their enhancers, with highly expressed eRNAs co-occurring with high expression of the enhancer target genes. Most eRNAs are not classified as a lncRNA due to their transient nature. Some enhancer transcription produces a stable, functional transcript akin to a lncRNA, though de-coupling the role of the act of transcription from activity of the RNA transcript itself has proven challenging [108]. Nevertheless, all eRNAs, at a minimum, exist transiently as nascent RNAs that can influence the surrounding chromatin from which they are produced, in some cases due to m6A methylation.

One of the first studies to identify m6A on eRNAs in 2019 found that a high portion of eRNAs had m6A modifications present [109]. A series of studies has led to an emerging model that the presence m6A increases the stability of many specific eRNAs [18,61,63,108]. In a study that used a high-sensitivity MINT-seq technique to identify m6A on nascent transcripts, m6A on eRNAs was found to facilitate transcriptional condensate formation and gene activation. Here, m6A methylation was shown to be enriched on long eRNAs and to enable interactions with YTHDC1, leading to transcriptional condensate formation involving recruitment of and co-mixing with BRD4 condensates to promote active transcription [20,62]. In a 2022 study that examined m6A on nascent transcripts using PRO-seq, m6A modification was identified on pre-mRNAs, promoter upstream transcripts, and eRNAs, and these transcripts were significantly depleted upon METTL3 knockdown. The results of this study suggested that m6A modification protects nascent RNAs from transcription termination by the Integrator complex to promote productive transcription [110]. Taken together, these studies highlight that m6A on eRNAs is important for their stability, thereby helping them enhance transcription at their paired promoters.

## 7. Phase Separation in the Context of m6A and LncRNA Biology

The formation of biomolecular condensates is driven by the process of liquid–liquid phase separation (LLPS) in which molecules spontaneously generate dense compartments, termed membraneless organelles, with enriched concentrations of specific proteins and RNAs. These can form in both the cytoplasm (stress granules, processing bodies (P-bodies)) or nucleus (nucleolus, nuclear speckles) and have diverse functions in various cellular processes [111]. Multivalent interactions between proteins and RNA molecules contribute to the ability to form condensates, with increased valency contributing to the strength of LLPS [112,113,114,115].

With RNA being a major component of biomolecular condensates, how RNA modifications might regulate phase separation is an important consideration. For example, m6A on mRNA contributes to stress granule and P-body formation in the cytoplasm, which is mediated by the binding of the cytoplasmic m6A readers YTHDF1-3 and IGF2BP3 that contain disordered domains that drive LLPS of the reader proteins and their m6A-modified targets [116,117,118,119,120,121,122]. m6A on mRNA can also facilitate phase separation in the nucleus, with one study in acute myeloid leukemia (AML) demonstrating that YTHDC1-m6A nuclear condensates are increased in AML cells compared to normal hematopoietic stem cells. These condensates act to protect oncogenic m6A-modified mRNAs from degradation, enabling cancer cell survival [123]. This nuclear condensation of methylated mRNAs by YTHDC1 also has implications for m6A-modified lncRNAs which tend to be localized in the nucleus. In addition to LLPS of the m6A reader proteins, components of the m6A methyltransferase complex have also been found to undergo phase separation [124,125,126,127].

The ability of lncRNAs to drive phase-separated condensate formation is of note, especially in the nucleus. Examples include Xist-mediated Barr body formation, nuclear-enriched abundant transcript 1 isoform 2 (*NEAT1_2*)-mediated paraspeckles, human satellite III (HSATIII)-mediated nuclear stress bodies, and enhancer RNA (eRNA)-mediated transcriptional condensates [128,129,130,131]. Recent studies demonstrate that m6A modification and YTHDC1 are involved in the mechanism by which these lncRNAs regulate the formation of condensates.

### 7.1. Xist Condensation of the Inactive X

As previously discussed, Xist is the lncRNA master regular of XCI, a process that involves the silencing and condensation of the inactive X chromosome during the development of female mammals. The formation of this condensed X chromosome is suggested to be a repressive form of phase separation that can be visualized by microscopy as a dense mass on the periphery of the nucleus [128]. The E-repeat-element of Xist recruits a multiprotein assembly that mediates condensate formation of the inactive X via self-aggregation and heterotypic protein interactions. This condensate is required for sustained gene silencing and anchoring of *Xist* to the inactive X, as well as maintenance of X-chromosome inactivation in the absence of *Xist* [132]. With the presence of over 60 m6A sites on Xist enabling the docking of multiple YTHDC1 reader proteins, the scaffolding ability of Xist to localize several YTHDC1 proteins together supports a model where multivalent interactions can drive phase separation of the entire X chromosome [82]. With the ability of Xist to induce heterochromatin of an entire X chromosome, it is possible that other lncRNAs that require m6A to induce chromatin silencing (i.e., HOTAIR, LINEs) may also employ repressive phase separation to facilitate this process.

### 7.2. NEAT1 and Paraspeckle Formation

Paraspeckles are membraneless nuclear bodies found near nuclear speckles in mammalian nuclei. The 23 kb long lncRNA *NEAT1* isoform 2 (NEAT1_2) is a scaffold component of paraspeckles, residing at their core and driving their formation, via the localized high concentration of nascent NEAT1_2 transcripts, which induce phase separation [133]. Containing over 60 identified protein components, paraspeckles are complex compartments within the nucleus that regulate processes such as RNA metabolism, gene expression, and DNA damage response [134]. NEAT1 is overexpressed in many tumors, suggesting a role in cancer [135].

**Figure 4:**
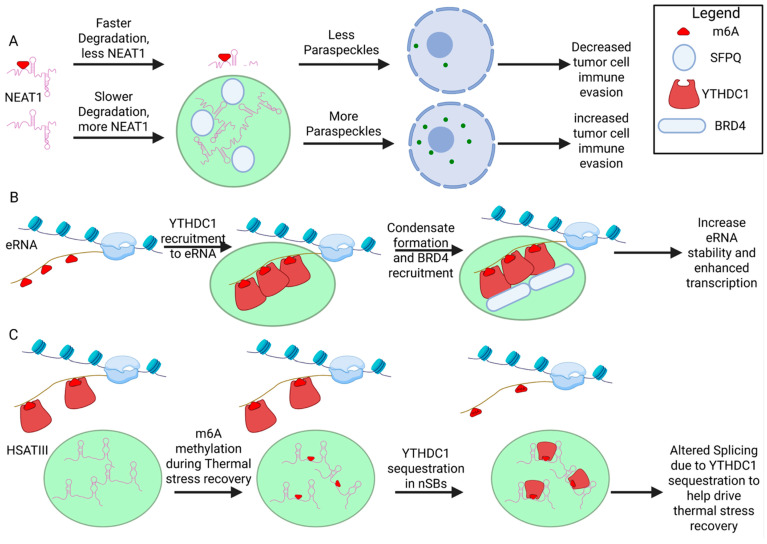
**Role of liquid–liquid phase separation (LLPS) in m6A mechanisms.** (**A**) The lncRNA NEAT1 is a scaffold component of paraspeckles and contributes to their formation. When NEAT1 undergoes m6A methylation, the lncRNA is more rapidly degraded, leading to reduced paraspeckle formation. Conversely, when NEAT1 does not have m6A methylation, its expression is increased, leading to more paraspeckles forming. (**B**) m6A methylation on eRNAs recruits YTHDC1. YTHDC1 IDRs then help drive LLPS, recruiting BRD4 condensates to help enhance transcription. (**C**) HSATIII undergoes m6A methylation in nuclear stress bodies (nSBs), leading to the sequestration of YTHDC1 into nSBs and altered splicing. Created in BioRender. Gonzalez, S. (2025) https://BioRender.com/4ifpmdu (accedd on 21 November 2025).

Several studies have demonstrated a role for m6A in regulating *NEAT1* function in cancer contexts. For example, NEAT1 accumulates at DNA double-strand breaks in human U2OS osteosarcoma cells to facilitate genome repair, and this is dependent upon METTL3-mediated m6A modification [136]. The m6A demethylase ALKBH5 acts to demethylate NEAT1 in gastric and colon cancer, leading to increased NEAT1 expression and cancer malignancy [137,138]. Similarly, in glioblastoma multiforme (GBM), hypoxia-induced ALKBH5 is upregulated and demethylates NEAT1, stabilizing it and increasing paraspeckle formation. This induces relocalization of the transcriptional repressor SFPQ from the CXCL8 promoter to paraspeckles, leading to an immunosuppressive tumor microenvironment and immune evasion [139]. Interestingly, the intrinsically disordered C terminal domain of ALKBH5 is required to drive its incorporation into paraspeckles and increase paraspeckles in response to hypoxia via demethylation and stabilization of NEAT1 [129]. Altogether, these studies suggest a key function for the m6A modification of NEAT1 in paraspeckle formation and dynamics (Figure 4A), with unmethylated NEAT1 accumulating in and increasing formation of paraspeckles leading to increased cancer malignancy, and methylated NEAT1 functioning at DNA double-strand breaks to facilitate genome repair.

### 7.3. eRNA-Mediated Transcriptional Condensates

As previously discussed, enhancer RNAs (eRNAs) are modified by the m6A methyltransferase complex [62,110]. This modification helps promote the formation of transcriptional condensates that form at active sites of transcription by incorporating transcription factors, co-activators, core transcriptional machinery, eRNAs, and underlying chromatin [130]. Unlike repressive phase-separated compartments, transcriptional condensates drive active transcription by forming hubs of transcriptionally active clusters of enhancers and their target genes. eRNA m6A methylation is enriched on long eRNAs and enables interaction with nuclear m6A reader YTHDC1 leading to the formation of YTHDC1 condensates. Condensate formation is enabled by the arginine residues in the YTHDC1 intrinsically disordered region 2, which support the co-mixing and augmentation of BRD4 condensates (Figure 4B) [62].

### 7.4. HSATIII and Nuclear Stress Bodies

Nuclear stress bodies (nSBs) are membraneless organelles formed in the nucleus upon thermal stress. Their formation is dependent upon the architectural lncRNAs HSATIII, primate-specific transcripts produced from pericentromeric satellite III regions on several chromosomes [131]. nSBs act to selectively sequester serine/arginine-rich splicing factors (SRSFs) during thermal stress to enable the retention of introns during stress recovery [140]. The m6A methyltransferase complex is recruited to nSBs during thermal stress recovery, leading to high levels of m6A modification on HSATIII lncRNAs [141]. This results in the sequestration of YTHDC1 to nSBs, depleting it from the nucleoplasm and preventing the splicing of introns regulated by the HSATIII lncRNA (Figure 4C). The m6A modification maps to the noncanonical m6A motif GGAAU repeat sequence in HSATIII, which is believed to be accomplished via enrichment of the m6A methyltransferase complex within nSBs to subvert the normal requisite cytosine downstream of the methylated adenosine. Multiple GGAAU repeat sequences present in HSATIII bind SRSF9 while m6A-modified GGAAU instead binds YTHDC1, leading to the formation of distinct ribonucleoprotein complexes that cooperatively control intron retention during thermal stress recovery.

### 7.5. Other Examples of m6A and ncRNA Involvement in LLPS

Several additional examples of m6A driving the phase separation of lncRNAs exist. These include lncRNA RUNX-IT1 driving the phase separation of the m6A reader IGF2BP1 to increase the mRNA stability of GPX4 [142], m6A modification of TERRA lncRNA mediating telomere stability and R-loop formation [143,144], centromeric RNA (cenRNA) interactions with the chromosome passenger complex (CPC) and a role for cenRNA m6A modification in promoting centromere integrity in cancer cells [145,146,147], and lncNONMMUT062668.2 m6A facilitating YTHDC1 phase separation, driving its nuclear export to exacerbate pulmonary fibrosis [148].

Overall, m6A on lncRNAs enables LLPS in a context-dependent manner. From repressive condensate formation of the X chromosome by Xist to active condensate formation by eRNAs at transcriptional hubs, m6A on noncoding RNA transcripts can drive interactions with proteins to enable biomolecular condensate formation which exerts an impact on the RNAs, chromatin, and proteins involved. Frequently, m6A-dependent condensate formation involves recruitment of YTHDC1, although other components of the m6A machinery, including components of the methyltransferase complex and demethylase ALKBH5, can also undergo and contribute to m6A-mediated phase separation [124,129]. Additional research will be important to elucidate the breadth and rules of m6A’s potential in regulating the phase separation of lncRNAs.

## 8. Cytoplasmic m6A-Modified LncRNAs

Most of the work in the literature looking at cytoplasmic m6A methylation focuses on mRNAs, rather than lncRNAs; however, some examples exist of m6A-regulated lncRNAs in the cytoplasm. One such lncRNA is THOR, which stands for testis-associated highly conserved oncogenic long non-coding RNA. THOR interacts with IGF2BP1 to help stabilize m6A-modified mRNAs in the cytoplasm via an unknown mechanism [149,150]. The THOR lncRNA has a shorter half-life in the cytoplasm when m6A-modified [149]. THOR has lower expression when the cytoplasmic reader YTHDF1 is knocked down but greater expression when its other reader YTHDF2 is knocked down, demonstrating differential effects of m6A on RNA stability in the cytoplasm based on the m6A reader [149]. These two readers are thought to bind different m6A sites on THOR, suggesting differential m6A effects on cytoplasmic lncRNAs depending on the RNA sequence context. The cytoplasmic lncRNA DANCR, which is involved in cancer stemness [151], undergoes m6A methylation at site A664, thereby recruiting IGF2BP2 [152]. IGF2BP2 then helps increase the stability of DANCR, allowing DANCR to more robustly support tumor progression [61,152]. One way DANCR increases tumor progression is by inhibiting microRNA (miRNA) binding to mRNAs either by directly sponging the miRNAs or by binding the mRNAs at the miRNA binding sites; both scenarios lead to increased stability of the mRNA and subsequent increased protein expression [153]. This is exemplified by DANCR acting as a sink for miRNAs that target genes like Nanog, Sox2, and Oct4, leading to higher expression of these genes and subsequent increased epithelial to mesenchymal transition (EMT) [153]. This example highlights how m6A modification of DANCR in the cytoplasm can have a direct impact on disease; in both acute myeloid leukemia and pancreatic cancer, increased stability of DANCR driven by IGF2BP binding m6A sites on DANCR drives disease progression. Recent studies have examined the effect of m6A methylation on the cytoplasmic lncRNA NORAD in intervertebral disks and in mesenchymal stem cells [154,155]. NORAD acts as a sponge for either miRNAs or proteins like Pumilio, helping increase the stability of mRNA transcripts normally degraded via these miRNAs or Pumilio [156]. In both cases, the presence of m6A on NORAD leads to reduced stability of the transcript, which appears to be YTHDF2-dependent, leading to more miRNA or Pumilio being available to inhibit the downstream mRNA normally protected by the presence of NORAD [154,155]. In turn, this led to altered disease progression, such as the senescence of nucleus pulposus cells in intervertebral discs and subsequent degeneration of intervertebral discs [154,155]. In each of these cases, the presence or absence of m6A on lncRNAs affects the progression of different diseases, highlighting the importance of understanding molecularly how these m6A methylation marks alter lncRNA biology.

While only a few examples, taken together, these studies also highlight how multiple m6A sites on one transcript can have differential effects depending on which m6A reader is recruited. Current work suggests the change in stability is caused by the recruitment of secondary proteins/complexes via interacting with the m6A reader. For example, YTHDF2 has been characterized to decrease stability via the recruitment of the CCR4-not complex, while IGF2BP2 increases stability by recruiting RNA stabilizing proteins like PABPC1. Furthermore, it is possible that changes to stability, while easily detectable, may not be the only function through which these m6A marks are altering molecular lncRNA mechanisms in the cytoplasm, even in the absence of translational control. There is much work still to be done to determine how cytoplasmic lncRNAs are influenced by m6A methylation as an additional step of regulation of their molecular mechanism.

## 9. Conclusions

As this review highlights, the diverse regulatory mechanisms that are triggered by m6A modification are clearly harnessed and integrated in many ways into lncRNA biology. While some patterns have emerged, one theme that persists is that context dictates outcome. The combination of a specific RNA transcript and the specific nucleotide that is methylated, the subcellular location or chromatin context of the RNA, and interaction with distinct readers that recruit additional distinct effector proteins, determines which molecular mechanism is initiated and how that will impact the greater cellular and organismal biology. Future work incorporating new methods for absolute m6A quantification, screening for m6A reader–effector relationships, and RNA structural analysis in living cells, will continue to contribute in rigorous ways to understanding at the molecular level. Additionally, precise genetic perturbation of the many lncRNA m6A sites will test their importance more directly. It is an exciting time, as these mechanisms are becoming more fully explored to gain insight into the manner in which epitranscriptomics can tune noncoding RNA function.

## Data Availability

Not applicable.

## Supplementary Materials

The following supporting information can be downloaded at: https://www.mdpi.com/article/10.3390/ncrna12010004/s1, Table S1: List of m6A-modified lncRNAs, the readers that bind them, and biological outcome of the interaction.

## References

[1] Mudge J.M., Carbonell-Sala S., Diekhans M., Martinez J.G., Hunt T., Jungreis I., Loveland J.E., Arnan C., Barnes I., Bennett R. (2025). GENCODE 2025: Reference gene annotation for human and mouse. Nucleic Acids Res..

[2] Porman A.M., Roberts J.T., Duncan E.D., Chrupcala M.L., Levine A.A., Kennedy M.A., Williams M.M., Richer J.K., Johnson A.M. (2022). A single N6-methyladenosine site regulates lncRNA HOTAIR function in breast cancer cells. PLoS Biol..

[3] Ke S., Pandya-Jones A., Saito Y., Fak J.J., Vagbo C.B., Geula S., Hanna J.H., Black D.L., Darnell J.E., Darnell R.B. (2017). m(6)A mRNA modifications are deposited in nascent pre-mRNA and are not required for splicing but do specify cytoplasmic turnover. Genes Dev..

[4] Knuckles P., Lence T., Haussmann I.U., Jacob D., Kreim N., Carl S.H., Masiello I., Hares T., Villasenor R., Hess D. (2018). Zc3h13/Flacc is required for adenosine methylation by bridging the mRNA-binding factor Rbm15/Spenito to the m(6)A machinery component Wtap/Fl(2)d. Genes Dev..

[5] Horiuchi K., Kawamura T., Iwanari H., Ohashi R., Naito M., Kodama T., Hamakubo T. (2013). Identification of Wilms’ tumor 1-associating protein complex and its role in alternative splicing and the cell cycle. J. Biol. Chem..

[6] He P.C., He C. (2021). m(6) A RNA methylation: From mechanisms to therapeutic potential. EMBO J..

[7] Liu J., Yue Y., Han D., Wang X., Fu Y., Zhang L., Jia G., Yu M., Lu Z., Deng X. (2013). A METTL3-METTL14 complex mediates mammalian nuclear RNA N6-adenosine methylation. Nat. Chem. Biol..

[8] Ruszkowska A., Ruszkowski M., Dauter Z., Brown J.A. (2018). Structural insights into the RNA methyltransferase domain of METTL16. Sci. Rep..

[9] Doxtader K.A., Wang P., Scarborough A.M., Seo D., Conrad N.K., Nam Y. (2018). Structural Basis for Regulation of METTL16, an S-Adenosylmethionine Homeostasis Factor. Mol. Cell.

[10] Meiser N., Mench N., Hengesbach M. (2020). RNA secondary structure dependence in METTL3-METTL14 mRNA methylation is modulated by the N-terminal domain of METTL3. Biol. Chem..

[11] Yang X., Triboulet R., Liu Q., Sendinc E., Gregory R.I. (2022). Exon junction complex shapes the m(6)A epitranscriptome. Nat. Commun..

[12] Uzonyi A., Dierks D., Nir R., Kwon O.S., Toth U., Barbosa I., Burel C., Brandis A., Rossmanith W., Le Hir H. (2023). Exclusion of m6A from splice-site proximal regions by the exon junction complex dictates m6A topologies and mRNA stability. Mol. Cell.

[13] Luo Z., Ma Q., Sun S., Li N., Wang H., Ying Z., Ke S. (2023). Exon-intron boundary inhibits m(6)A deposition, enabling m(6)A distribution hallmark, longer mRNA half-life and flexible protein coding. Nat. Commun..

[14] He P.C., Wei J., Dou X., Harada B.T., Zhang Z., Ge R., Liu C., Zhang L.S., Yu X., Wang S. (2023). Exon architecture controls mRNA m(6)A suppression and gene expression. Science.

[15] Guerrero-Castillo S., Cabrera-Orefice A., Huynen M.A., Arnold S. (2017). Identification and evolutionary analysis of tissue-specific isoforms of mitochondrial complex I subunit NDUFV3. Biochim. Biophys. Acta Bioenerg..

[16] Gao Z., Zha X., Li M., Xia X., Wang S. (2024). Insights into the m(6)A demethylases FTO and ALKBH5: Structural, biological function, and inhibitor development. Cell Biosci..

[17] Toh J.D.W., Crossley S.W.M., Bruemmer K.J., Ge E.J., He D., Iovan D.A., Chang C.J. (2020). Distinct RNA N-demethylation pathways catalyzed by nonheme iron ALKBH5 and FTO enzymes enable regulation of formaldehyde release rates. Proc. Natl. Acad. Sci. USA.

[18] Wei J., Liu F., Lu Z., Fei Q., Ai Y., He P.C., Shi H., Cui X., Su R., Klungland A. (2018). Differential m(6)A, m(6)A(m), and m(1)A Demethylation Mediated by FTO in the Cell Nucleus and Cytoplasm. Mol. Cell.

[19] Yu F., Zhu A.C., Liu S., Gao B., Wang Y., Khudaverdyan N., Yu C., Wu Q., Jiang Y., Song J. (2023). RBM33 is a unique m(6)A RNA-binding protein that regulates ALKBH5 demethylase activity and substrate selectivity. Mol. Cell.

[20] Li R., Zhao H., Huang X., Zhang J., Bai R., Zhuang L., Wen S., Wu S., Zhou Q., Li M. (2023). Super-enhancer RNA m(6)A promotes local chromatin accessibility and oncogene transcription in pancreatic ductal adenocarcinoma. Nat. Genet..

[21] Liao S., Sun H., Xu C. (2018). YTH Domain: A Family of N(6)-methyladenosine (m(6)A) Readers. Genom. Proteom. Bioinform..

[22] Li F., Zhao D., Wu J., Shi Y. (2014). Structure of the YTH domain of human YTHDF2 in complex with an m(6)A mononucleotide reveals an aromatic cage for m(6)A recognition. Cell Res..

[23] Sikorski V., Selberg S., Lalowski M., Karelson M., Kankuri E. (2023). The structure and function of YTHDF epitranscriptomic m(6)A readers. Trends Pharmacol. Sci..

[24] Dai X., Wang T., Gonzalez G., Wang Y. (2018). Identification of YTH Domain-Containing Proteins as the Readers for N1-Methyladenosine in RNA. Anal. Chem..

[25] Petri B.J., Klinge C.M. (2023). m6A readers, writers, erasers, and the m6A epitranscriptome in breast cancer. J. Mol. Endocrinol..

[26] Nicastro G., Abis G., Klein P., Esteban-Serna S., Gallagher C., Chaves-Arquero B., Cai Y., Figueiredo A.M., Martin S.R., Patani R. (2023). Direct m6A recognition by IMP1 underlays an alternative model of target selection for non-canonical methyl-readers. Nucleic Acids Res..

[27] Fakhar M., Gul M., Li W. (2024). Interactive Structural Analysis of KH3-4 Didomains of IGF2BPs with Preferred RNA Motif Having m(6)A Through Dynamics Simulation Studies. Int. J. Mol. Sci..

[28] Flamand M.N., Tegowski M., Meyer K.D. (2023). The Proteins of mRNA Modification: Writers, Readers, and Erasers. Annu. Rev. Biochem..

[29] Krecic A.M., Swanson M.S. (1999). hnRNP complexes: Composition, structure, and function. Curr. Opin. Cell Biol..

[30] Wu B., Su S., Patil D.P., Liu H., Gan J., Jaffrey S.R., Ma J. (2018). Molecular basis for the specific and multivariant recognitions of RNA substrates by human hnRNP A2/B1. Nat. Commun..

[31] Geuens T., Bouhy D., Timmerman V. (2016). The hnRNP family: Insights into their role in health and disease. Hum. Genet..

[32] Li F., Li W. (2024). Readers of RNA Modification in Cancer and Their Anticancer Inhibitors. Biomolecules.

[33] Hou J., Gao Y., Han B., Yan S., Wei S., Gao X. (2025). Nuclear accumulation of YTHDF1 regulates mRNA splicing in the DNA damage response. Sci. Adv..

[34] Zhou J., Wan J., Gao X., Zhang X., Jaffrey S.R., Qian S.B. (2015). Dynamic m(6)A mRNA methylation directs translational control of heat shock response. Nature.

[35] Kirli K., Karaca S., Dehne H.J., Samwer M., Pan K.T., Lenz C., Urlaub H., Gorlich D. (2015). A deep proteomics perspective on CRM1-mediated nuclear export and nucleocytoplasmic partitioning. eLife.

[36] Li J., Ahmad M., Sang L., Zhan Y., Wang Y., Yan Y., Liu Y., Mi W., Lu M., Dai Y. (2023). O-GlcNAcylation promotes the cytosolic localization of the m(6)A reader YTHDF1 and colorectal cancer tumorigenesis. J. Biol. Chem..

[37] Wachter K., Kohn M., Stohr N., Huttelmaier S. (2013). Subcellular localization and RNP formation of IGF2BPs (IGF2 mRNA-binding proteins) is modulated by distinct RNA-binding domains. Biol. Chem..

[38] Rivera Vargas T., Boudoukha S., Simon A., Souidi M., Cuvellier S., Pinna G., Polesskaya A. (2014). Post-transcriptional regulation of cyclins D1, D3 and G1 and proliferation of human cancer cells depend on IMP-3 nuclear localization. Oncogene.

[39] Sterky F.H., Ruzzenente B., Gustafsson C.M., Samuelsson T., Larsson N.G. (2010). LRPPRC is a mitochondrial matrix protein that is conserved in metazoans. Biochem. Biophys. Res. Commun..

[40] Nichols R.C., Wang X.W., Tang J., Hamilton B.J., High F.A., Herschman H.R., Rigby W.F. (2000). The RGG domain in hnRNP A2 affects subcellular localization. Exp. Cell Res..

[41] Lo J., Vaeth K.F., Bhardwaj G., Mukherjee N., Russ H.A., Moore J.K., Taliaferro J.M. (2024). The RNA binding protein HNRNPA2B1 regulates RNA abundance and motor protein activity in neurites. bioRxiv.

[42] Martino F., Varadarajan N.M., Perestrelo A.R., Hejret V., Durikova H., Vukic D., Horvath V., Cavalieri F., Caruso F., Albihlal W.S. (2022). The mechanical regulation of RNA binding protein hnRNPC in the failing heart. Sci. Transl. Med..

[43] Xu C., Wang X., Liu K., Roundtree I.A., Tempel W., Li Y., Lu Z., He C., Min J. (2014). Structural basis for selective binding of m6A RNA by the YTHDC1 YTH domain. Nat. Chem. Biol..

[44] Arguello A.E., Leach R.W., Kleiner R.E. (2019). In Vitro Selection with a Site-Specifically Modified RNA Library Reveals the Binding Preferences of N(6)-Methyladenosine Reader Proteins. Biochemistry.

[45] Liu B., Merriman D.K., Choi S.H., Schumacher M.A., Plangger R., Kreutz C., Horner S.M., Meyer K.D., Al-Hashimi H.M. (2018). A potentially abundant junctional RNA motif stabilized by m(6)A and Mg(2). Nat. Commun..

[46] Lu S., Han L., Hu X., Sun T., Xu D., Li Y., Chen Q., Yao W., He M., Wang Z. (2021). N6-methyladenosine reader IMP2 stabilizes the ZFAS1/OLA1 axis and activates the Warburg effect: Implication in colorectal cancer. J. Hematol. Oncol..

[47] Liu N., Dai Q., Zheng G., He C., Parisien M., Pan T. (2015). N(6)-methyladenosine-dependent RNA structural switches regulate RNA-protein interactions. Nature.

[48] Xiong X., Feng S., Ma X., Liu K., Gui Y., Chen B., Fan X., Wang F., Wang X., Yuan S. (2025). hnRNPC Functions with HuR to Regulate Alternative Splicing in an m6A-Dependent Manner and is Essential for Meiosis. Adv. Sci..

[49] Kumar A., Daripa P., Penumutchu S., Maiti S., Jain N. (2025). Thermodynamic insights into N6-methyladenosine-modified ribonucleic acids and their interactions with the RNA recognition motif of heterogeneous nuclear ribonucleoprotein C. Int. J. Biol. Macromol..

[50] Liu N., Zhou K.I., Parisien M., Dai Q., Diatchenko L., Pan T. (2017). N6-methyladenosine alters RNA structure to regulate binding of a low-complexity protein. Nucleic Acids Res..

[51] Wang Y., Li Y., Toth J.I., Petroski M.D., Zhang Z., Zhao J.C. (2014). N6-methyladenosine modification destabilizes developmental regulators in embryonic stem cells. Nat. Cell Biol..

[52] Spahr H., Rozanska A., Li X., Atanassov I., Lightowlers R.N., Chrzanowska-Lightowlers Z.M., Rackham O., Larsson N.G. (2016). SLIRP stabilizes LRPPRC via an RRM-PPR protein interface. Nucleic Acids Res..

[53] Roost C., Lynch S.R., Batista P.J., Qu K., Chang H.Y., Kool E.T. (2015). Structure and thermodynamics of N6-methyladenosine in RNA: A spring-loaded base modification. J. Am. Chem. Soc..

[54] Zhou K.I., Parisien M., Dai Q., Liu N., Diatchenko L., Sachleben J.R., Pan T. (2016). N(6)-Methyladenosine Modification in a Long Noncoding RNA Hairpin Predisposes Its Conformation to Protein Binding. J. Mol. Biol..

[55] Wei G. (2024). RNA m6A modification, signals for degradation or stabilisation?. Biochem. Soc. Trans..

[56] Huang H., Weng H., Sun W., Qin X., Shi H., Wu H., Zhao B.S., Mesquita A., Liu C., Yuan C.L. (2018). Recognition of RNA N(6)-methyladenosine by IGF2BP proteins enhances mRNA stability and translation. Nat. Cell Biol..

[57] Boo S.H., Kim Y.K. (2020). The emerging role of RNA modifications in the regulation of mRNA stability. Exp. Mol. Med..

[58] Wang X., Lu Z., Gomez A., Hon G.C., Yue Y., Han D., Fu Y., Parisien M., Dai Q., Jia G. (2014). N6-methyladenosine-dependent regulation of messenger RNA stability. Nature.

[59] Du H., Zhao Y., He J., Zhang Y., Xi H., Liu M., Ma J., Wu L. (2016). YTHDF2 destabilizes m(6)A-containing RNA through direct recruitment of the CCR4-NOT deadenylase complex. Nat. Commun..

[60] Park O.H., Ha H., Lee Y., Boo S.H., Kwon D.H., Song H.K., Kim Y.K. (2019). Endoribonucleolytic Cleavage of m(6)A-Containing RNAs by RNase P/MRP Complex. Mol. Cell.

[61] Wu S., Chi C., Weng S., Zhou W., Liu Z. (2023). IGF2BP2 promotes lncRNA DANCR stability mediated glycolysis and affects the progression of FLT3-ITD + acute myeloid leukemia. Apoptosis.

[62] Lee J.H., Wang R., Xiong F., Krakowiak J., Liao Z., Nguyen P.T., Moroz-Omori E.V., Shao J., Zhu X., Bolt M.J. (2021). Enhancer RNA m6A methylation facilitates transcriptional condensate formation and gene activation. Mol. Cell.

[63] Liang D., Lin W.J., Ren M., Qiu J., Yang C., Wang X., Li N., Zeng T., Sun K., You L. (2022). m(6)A reader YTHDC1 modulates autophagy by targeting SQSTM1 in diabetic skin. Autophagy.

[64] Zhang Z., Wang Q., Zhao X., Shao L., Liu G., Zheng X., Xie L., Zhang Y., Sun C., Xu R. (2020). YTHDC1 mitigates ischemic stroke by promoting Akt phosphorylation through destabilizing PTEN mRNA. Cell Death Dis..

[65] Liu J., Dou X., Chen C., Chen C., Liu C., Xu M.M., Zhao S., Shen B., Gao Y., Han D. (2020). N (6)-methyladenosine of chromosome-associated regulatory RNA regulates chromatin state and transcription. Science.

[66] Wei G., Coker H., Rodermund L., Almeida M., Roach H.L., Nesterova T.B., Brockdorff N. (2025). m(6)A and the NEXT complex direct Xist RNA turnover and X-inactivation dynamics. Nat. Struct. Mol. Biol..

[67] Saglam B., Akgul B. (2024). An Overview of Current Detection Methods for RNA Methylation. Int. J. Mol. Sci..

[68] Xiao Y.L., Liu S., Ge R., Wu Y., He C., Chen M., Tang W. (2023). Transcriptome-wide profiling and quantification of N(6)-methyladenosine by enzyme-assisted adenosine deamination. Nat. Biotechnol..

[69] Zhong Z.D., Xie Y.Y., Chen H.X., Lan Y.L., Liu X.H., Ji J.Y., Wu F., Jin L., Chen J., Mak D.W. (2023). Systematic comparison of tools used for m(6)A mapping from nanopore direct RNA sequencing. Nat. Commun..

[70] Sun H., Lu B., Zhang Z., Xiao Y., Zhou Z., Xi L., Li Z., Jiang Z., Zhang J., Wang M. (2025). Mild and ultrafast GLORI enables absolute quantification of m(6)A methylome from low-input samples. Nat. Methods.

[71] Linder B., Grozhik A.V., Olarerin-George A.O., Meydan C., Mason C.E., Jaffrey S.R. (2015). Single-nucleotide-resolution mapping of m6A and m6Am throughout the transcriptome. Nat. Methods.

[72] Chen K., Lu Z., Wang X., Fu Y., Luo G.Z., Liu N., Han D., Dominissini D., Dai Q., Pan T. (2015). High-resolution N(6) -methyladenosine (m(6) A) map using photo-crosslinking-assisted m(6) A sequencing. Angew. Chem. Int. Ed. Engl..

[73] Shen W., Sun H., Liu C., Yi Y., Hou Y., Xiao Y., Hu Y., Lu B., Peng J., Wang J. (2024). GLORI for absolute quantification of transcriptome-wide m(6)A at single-base resolution. Nat. Protoc..

[74] Liu C., Sun H., Yi Y., Shen W., Li K., Xiao Y., Li F., Li Y., Hou Y., Lu B. (2023). Absolute quantification of single-base m(6)A methylation in the mammalian transcriptome using GLORI. Nat. Biotechnol..

[75] Brown C.J., Hendrich B.D., Rupert J.L., Lafreniere R.G., Xing Y., Lawrence J., Willard H.F. (1992). The human XIST gene: Analysis of a 17 kb inactive X-specific RNA that contains conserved repeats and is highly localized within the nucleus. Cell.

[76] Penny G.D., Kay G.F., Sheardown S.A., Rastan S., Brockdorff N. (1996). Requirement for Xist in X chromosome inactivation. Nature.

[77] Brockdorff N., Bowness J.S., Wei G. (2020). Progress toward understanding chromosome silencing by Xist RNA. Genes Dev..

[78] McHugh C.A., Chen C.K., Chow A., Surka C.F., Tran C., McDonel P., Pandya-Jones A., Blanco M., Burghard C., Moradian A. (2015). The Xist lncRNA interacts directly with SHARP to silence transcription through HDAC3. Nature.

[79] Chu C., Zhang Q.C., da Rocha S.T., Flynn R.A., Bharadwaj M., Calabrese J.M., Magnuson T., Heard E., Chang H.Y. (2015). Systematic discovery of Xist RNA binding proteins. Cell.

[80] Minajigi A., Froberg J.E., Wei C., Sunwoo H., Kesner B., Colognori D., Lessing D., Payer B., Boukhali M., Haas W. (2015). Chromosomes. A comprehensive Xist interactome reveals cohesin repulsion and an RNA-directed chromosome conformation. Science.

[81] Moindrot B., Cerase A., Coker H., Masui O., Grijzenhout A., Pintacuda G., Schermelleh L., Nesterova T.B., Brockdorff N. (2015). A Pooled shRNA Screen Identifies Rbm15, Spen, and Wtap as Factors Required for Xist RNA-Mediated Silencing. Cell Rep..

[82] Patil D.P., Chen C.K., Pickering B.F., Chow A., Jackson C., Guttman M., Jaffrey S.R. (2016). m(6)A RNA methylation promotes XIST-mediated transcriptional repression. Nature.

[83] Nesterova T.B., Wei G., Coker H., Pintacuda G., Bowness J.S., Zhang T., Almeida M., Bloechl B., Moindrot B., Carter E.J. (2019). Systematic allelic analysis defines the interplay of key pathways in X chromosome inactivation. Nat. Commun..

[84] Dossin F., Pinheiro I., Zylicz J.J., Roensch J., Collombet S., Le Saux A., Chelmicki T., Attia M., Kapoor V., Zhan Y. (2020). SPEN integrates transcriptional and epigenetic control of X-inactivation. Nature.

[85] Coker H., Wei G., Moindrot B., Mohammed S., Nesterova T., Brockdorff N. (2020). The role of the Xist 5’ m6A region and RBM15 in X chromosome inactivation. Wellcome Open Res..

[86] Lu Z., Guo J.K., Wei Y., Dou D.R., Zarnegar B., Ma Q., Li R., Zhao Y., Liu F., Choudhry H. (2020). Structural modularity of the XIST ribonucleoprotein complex. Nat. Commun..

[87] Wei G., Almeida M., Pintacuda G., Coker H., Bowness J.S., Ule J., Brockdorff N. (2021). Acute depletion of METTL3 implicates N (6)-methyladenosine in alternative intron/exon inclusion in the nascent transcriptome. Genome Res..

[88] Chang C., Ma G., Cheung E., Hutchins A.P. (2022). A programmable system to methylate and demethylate N(6)-methyladenosine (m(6)A) on specific RNA transcripts in mammalian cells. J. Biol. Chem..

[89] Rinn J.L., Kertesz M., Wang J.K., Squazzo S.L., Xu X., Brugmann S.A., Goodnough L.H., Helms J.A., Farnham P.J., Segal E. (2007). Functional demarcation of active and silent chromatin domains in human HOX loci by noncoding RNAs. Cell.

[90] Balas M.M., Johnson A.M. (2018). Exploring the mechanisms behind long noncoding RNAs and cancer. Non-Coding RNA Res..

[91] Gupta R.A., Shah N., Wang K.C., Kim J., Horlings H.M., Wong D.J., Tsai M.C., Hung T., Argani P., Rinn J.L. (2010). Long non-coding RNA HOTAIR reprograms chromatin state to promote cancer metastasis. Nature.

[92] Tsai M.C., Manor O., Wan Y., Mosammaparast N., Wang J.K., Lan F., Shi Y., Segal E., Chang H.Y. (2010). Long noncoding RNA as modular scaffold of histone modification complexes. Science.

[93] Somarowthu S., Legiewicz M., Chillon I., Marcia M., Liu F., Pyle A.M. (2015). HOTAIR forms an intricate and modular secondary structure. Mol. Cell.

[94] Meredith E.K., Balas M.M., Sindy K., Haislop K., Johnson A.M. (2016). An RNA matchmaker protein regulates the activity of the long noncoding RNA HOTAIR. RNA.

[95] Nguyen E.D., Balas M.M., Griffin A.M., Roberts J.T., Johnson A.M. (2018). Global profiling of hnRNP A2/B1-RNA binding on chromatin highlights LncRNA interactions. RNA Biol..

[96] Balas M.M., Hartwick E.W., Barrington C., Roberts J.T., Wu S.K., Bettcher R., Griffin A.M., Kieft J.S., Johnson A.M. (2021). Establishing RNA-RNA interactions remodels lncRNA structure and promotes PRC2 activity. Sci. Adv..

[97] Portoso M., Ragazzini R., Brencic Z., Moiani A., Michaud A., Vassilev I., Wassef M., Servant N., Sargueil B., Margueron R. (2017). PRC2 is dispensable for HOTAIR-mediated transcriptional repression. EMBO J..

[98] Delhaye L., De Bruycker E., Volders P.J., Fijalkowska D., De Sutter D., Degroeve S., Martens L., Mestdagh P., Eyckerman S. (2022). Orthogonal proteomics methods to unravel the HOTAIR interactome. Sci. Rep..

[99] Wu X.M., Mai Y.X., Wen Y.F., Li Z.P., Sun Y.X., Chen J.J., Meng F., Pang F.X., Li H.M., Pan Y. (2025). Silence of HOTAIR promotes osteogenic differentiation and accelerates distraction osteogenesis by mediating FTO ubiquitination. J. Orthop. Transl..

[100] Liu T.C., Li H.X., Wan Y.X., Shi G., Zhao Y.P., Liu Y.F., Fan X.Y. (2024). METTL14-mediated upregulation of lncRNA HOTAIR represses PP1alpha expression by promoting H3K4me1 demethylation in oxycodone-treated mice. CNS Neurosci. Ther..

[101] Liu J., Gao M., He J., Wu K., Lin S., Jin L., Chen Y., Liu H., Shi J., Wang X. (2021). The RNA m(6)A reader YTHDC1 silences retrotransposons and guards ES cell identity. Nature.

[102] Chen C., Liu W., Guo J., Liu Y., Liu X., Liu J., Dou X., Le R., Huang Y., Li C. (2021). Nuclear m(6)A reader YTHDC1 regulates the scaffold function of LINE1 RNA in mouse ESCs and early embryos. Protein Cell.

[103] Wei J., Yu X., Yang L., Liu X., Gao B., Huang B., Dou X., Liu J., Zou Z., Cui X.L. (2022). FTO mediates LINE1 m(6)A demethylation and chromatin regulation in mESCs and mouse development. Science.

[104] Xiong F., Wang R., Lee J.H., Li S., Chen S.F., Liao Z., Hasani L.A., Nguyen P.T., Zhu X., Krakowiak J. (2021). RNA m(6)A modification orchestrates a LINE-1-host interaction that facilitates retrotransposition and contributes to long gene vulnerability. Cell Res..

[105] Li Z., Fang F., Zafar M.I., Wu X., Liu X., Tan X., Luo J., Ye Z., Xiong C., Li H. (2024). RNA m(6)A modification regulates L1 retrotransposons in human spermatogonial stem cell differentiation in vitro and in vivo. Cell Mol. Life Sci..

[106] Chelmicki T., Roger E., Teissandier A., Dura M., Bonneville L., Rucli S., Dossin F., Fouassier C., Lameiras S., Bourc’his D. (2021). m(6)A RNA methylation regulates the fate of endogenous retroviruses. Nature.

[107] Altendorfer E., Mundlos S., Mayer A. (2025). A transcription coupling model for how enhancers communicate with their target genes. Nat. Struct. Mol. Biol..

[108] Ali T., Grote P. (2020). Beyond the RNA-dependent function of LncRNA genes. eLife.

[109] Xiao S., Cao S., Huang Q., Xia L., Deng M., Yang M., Jia G., Liu X., Shi J., Wang W. (2019). The RNA N(6)-methyladenosine modification landscape of human fetal tissues. Nat. Cell Biol..

[110] Xu W., He C., Kaye E.G., Li J., Mu M., Nelson G.M., Dong L., Wang J., Wu F., Shi Y.G. (2022). Dynamic control of chromatin-associated m(6)A methylation regulates nascent RNA synthesis. Mol. Cell.

[111] Banani S.F., Lee H.O., Hyman A.A., Rosen M.K. (2017). Biomolecular condensates: Organizers of cellular biochemistry. Nat. Rev. Mol. Cell Biol..

[112] Zumbro E., Alexander-Katz A. (2021). Multivalent polymers can control phase boundary, dynamics, and organization of liquid-liquid phase separation. PLoS ONE.

[113] Grese Z.R., Bastos A.C., Mamede L.D., French R.L., Miller T.M., Ayala Y.M. (2021). Specific RNA interactions promote TDP-43 multivalent phase separation and maintain liquid properties. EMBO Rep..

[114] Chen C., Jia H., Nakamura Y., Kanekura K., Hayamizu Y. (2022). Effect of Multivalency on Phase-Separated Droplets Consisting of Poly(PR) Dipeptide Repeats and RNA at the Solid/Liquid Interface. ACS Omega.

[115] Parker D.M., Tauber D., Parker R. (2025). G3BP1 promotes intermolecular RNA-RNA interactions during RNA condensation. Mol. Cell.

[116] Ries R.J., Zaccara S., Klein P., Olarerin-George A., Namkoong S., Pickering B.F., Patil D.P., Kwak H., Lee J.H., Jaffrey S.R. (2019). m(6)A enhances the phase separation potential of mRNA. Nature.

[117] Gao Y., Pei G., Li D., Li R., Shao Y., Zhang Q.C., Li P. (2019). Multivalent m(6)A motifs promote phase separation of YTHDF proteins. Cell Res..

[118] Wang J., Wang L., Diao J., Shi Y.G., Shi Y., Ma H., Shen H. (2020). Binding to m(6)A RNA promotes YTHDF2-mediated phase separation. Protein Cell.

[119] Liu S.Y., Feng Y., Wu J.J., Zou M.L., Sun Z.L., Li X., Yuan F.L. (2020). m(6) A facilitates YTHDF-independent phase separation. J. Cell. Mol. Med..

[120] Park J., Wu Y., Shao W., Gendron T.F., van der Spek S.J.F., Sultanakhmetov G., Basu A., Castellanos Otero P., Jones C.J., Jansen-West K. (2023). Poly(GR) interacts with key stress granule factors promoting its assembly into cytoplasmic inclusions. Cell Rep..

[121] Chen Y., Wan R., Zou Z., Lao L., Shao G., Zheng Y., Tang L., Yuan Y., Ge Y., He C. (2023). O-GlcNAcylation determines the translational regulation and phase separation of YTHDF proteins. Nat. Cell Biol..

[122] Shan T., Liu F., Wen M., Chen Z., Li S., Wang Y., Cheng H., Zhou Y. (2023). m(6)A modification negatively regulates translation by switching mRNA from polysome to P-body via IGF2BP3. Mol. Cell.

[123] Cheng Y., Xie W., Pickering B.F., Chu K.L., Savino A.M., Yang X., Luo H., Nguyen D.T., Mo S., Barin E. (2021). N(6)-Methyladenosine on mRNA facilitates a phase-separated nuclear body that suppresses myeloid leukemic differentiation. Cancer Cell.

[124] Han D., Longhini A.P., Zhang X., Hoang V., Wilson M.Z., Kosik K.S. (2022). Dynamic assembly of the mRNA m6A methyltransferase complex is regulated by METTL3 phase separation. PLoS Biol..

[125] Jiang A., Zhang S., Wang X., Li D. (2022). RBM15 condensates modulate m(6)A modification of STYK1 to promote tumorigenesis. Comput. Struct. Biotechnol. J..

[126] Ge Y., Chen R., Ling T., Liu B., Huang J., Cheng Y., Lin Y., Chen H., Xie X., Xia G. (2024). Elevated WTAP promotes hyperinflammation by increasing m6A modification in inflammatory disease models. J. Clin. Investig..

[127] Cai S., Zhou J., Luo X., Zhang C., Jin S., Ren J., Cui J. (2025). Phase transition of WTAP regulates m(6)A modification of interferon-stimulated genes. eLife.

[128] Cerase A., Armaos A., Neumayer C., Avner P., Guttman M., Tartaglia G.G. (2019). Phase separation drives X-chromosome inactivation: A hypothesis. Nat. Struct. Mol. Biol..

[129] Qin X., Long Y., Bai X., Cao L., Yan H., Zhang K., Wang B., Wu X. (2023). The disordered C terminus of ALKBH5 promotes phase separation and paraspeckles assembly. J. Biol. Chem..

[130] Demmerle J., Hao S., Cai D. (2023). Transcriptional condensates and phase separation: Condensing information across scales and mechanisms. Nucleus.

[131] Biamonti G., Vourc’h C. (2010). Nuclear stress bodies. Cold Spring Harb. Perspect. Biol..

[132] Pandya-Jones A., Markaki Y., Serizay J., Chitiashvili T., Mancia Leon W.R., Damianov A., Chronis C., Papp B., Chen C.K., McKee R. (2020). A protein assembly mediates Xist localization and gene silencing. Nature.

[133] Yamazaki T., Yamamoto T., Hirose T. (2022). Micellization: A new principle in the formation of biomolecular condensates. Front. Mol. Biosci..

[134] Yamazaki T., Hirose T. (2015). The building process of the functional paraspeckle with long non-coding RNAs. Front. Biosci. Elite Ed..

[135] Pisani G., Baron B. (2020). NEAT1 and Paraspeckles in Cancer Development and Chemoresistance. Noncoding RNA.

[136] Mamontova V., Trifault B., Gribling-Burrer A.S., Bohn P., Boten L., Preckwinkel P., Gallant P., Solvie D., Ade C.P., Papadopoulos D. (2024). NEAT1 promotes genome stability via m(6)A methylation-dependent regulation of CHD4. Genes Dev..

[137] Zhang J., Guo S., Piao H.Y., Wang Y., Wu Y., Meng X.Y., Yang D., Zheng Z.C., Zhao Y. (2019). ALKBH5 promotes invasion and metastasis of gastric cancer by decreasing methylation of the lncRNA NEAT1. J. Physiol. Biochem..

[138] Guo T., Liu D.F., Peng S.H., Xu A.M. (2020). ALKBH5 promotes colon cancer progression by decreasing methylation of the lncRNA NEAT1. Am. J. Transl. Res..

[139] Dong F., Qin X., Wang B., Li Q., Hu J., Cheng X., Guo D., Cheng F., Fang C., Tan Y. (2021). ALKBH5 Facilitates Hypoxia-Induced Paraspeckle Assembly and IL8 Secretion to Generate an Immunosuppressive Tumor Microenvironment. Cancer Res..

[140] Ninomiya K., Adachi S., Natsume T., Iwakiri J., Terai G., Asai K., Hirose T. (2020). LncRNA-dependent nuclear stress bodies promote intron retention through SR protein phosphorylation. EMBO J..

[141] Ninomiya K., Iwakiri J., Aly M.K., Sakaguchi Y., Adachi S., Natsume T., Terai G., Asai K., Suzuki T., Hirose T. (2021). m(6) A modification of HSATIII lncRNAs regulates temperature-dependent splicing. EMBO J..

[142] Wang S., Wang Y., Li Q., Zeng K., Li X., Feng X. (2023). RUNX1-IT1 favors breast cancer carcinogenesis through regulation of IGF2BP1/GPX4 axis. Discov. Oncol..

[143] Chen L., Zhang C., Ma W., Huang J., Zhao Y., Liu H. (2022). METTL3-mediated m6A modification stabilizes TERRA and maintains telomere stability. Nucleic Acids Res..

[144] Vaid R., Thombare K., Mendez A., Burgos-Panadero R., Djos A., Jachimowicz D., Lundberg K.I., Bartenhagen C., Kumar N., Tummler C. (2024). METTL3 drives telomere targeting of TERRA lncRNA through m6A-dependent R-loop formation: A therapeutic target for ALT-positive neuroblastoma. Nucleic Acids Res..

[145] Kang Z., Li R., Liu C., Dong X., Hu Y., Xu L., Liu X., Xiang Y., Gao L., Si W. (2024). m(6)A-modified cenRNA stabilizes CENPA to ensure centromere integrity in cancer cells. Cell.

[146] Blower M.D. (2016). Centromeric Transcription Regulates Aurora-B Localization and Activation. Cell Rep..

[147] Trivedi P., Palomba F., Niedzialkowska E., Digman M.A., Gratton E., Stukenberg P.T. (2019). The inner centromere is a biomolecular condensate scaffolded by the chromosomal passenger complex. Nat. Cell Biol..

[148] Chen S., Wang Y., Zhang J., Liu B., Liu W., Cao G., Li R., Li H., Zhai N., Song X. (2025). YTHDC1 phase separation drives the nuclear export of m(6)A-modified lncNONMMUT062668.2 through the transport complex SRSF3-ALYREF-XPO5 to aggravate pulmonary fibrosis. Cell Death Dis..

[149] Liu H., Xu Y., Yao B., Sui T., Lai L., Li Z. (2020). A novel N6-methyladenosine (m6A)-dependent fate decision for the lncRNA THOR. Cell Death Dis..

[150] Hosono Y., Niknafs Y.S., Prensner J.R., Iyer M.K., Dhanasekaran S.M., Mehra R., Pitchiaya S., Tien J., Escara-Wilke J., Poliakov A. (2023). Oncogenic Role of THOR, a Conserved Cancer/Testis Long Non-coding RNA. Cell.

[151] Thin K.Z., Liu X., Feng X., Raveendran S., Tu J.C. (2018). LncRNA-DANCR: A valuable cancer related long non-coding RNA for human cancers. Pathol. Res. Pract..

[152] Hu X., Peng W.X., Zhou H., Jiang J., Zhou X., Huang D., Mo Y.Y., Yang L. (2020). IGF2BP2 regulates DANCR by serving as an N6-methyladenosine reader. Cell Death Differ..

[153] Wang M., Gu J., Zhang X., Yang J., Zhang X., Fang X. (2021). Long Non-coding RNA DANCR in Cancer: Roles, Mechanisms, and Implications. Front. Cell Dev. Biol..

[154] Li G., Ma L., He S., Luo R., Wang B., Zhang W., Song Y., Liao Z., Ke W., Xiang Q. (2022). WTAP-mediated m(6)A modification of lncRNA NORAD promotes intervertebral disc degeneration. Nat. Commun..

[155] Liu W.J., Wang J.X., Li Q.F., Zhang Y.H., Ji P.F., Jin J.H., Zhang Y.B., Yuan Z.H., Feng P., Wu Y.F. (2025). Fat mass and obesity-associated protein in mesenchymal stem cells inhibits osteoclastogenesis via lnc NORAD/miR-4284 axis in ankylosing spondylitis. World J. Stem Cells.

[156] Soghli N., Yousefi T., Abolghasemi M., Qujeq D. (2021). NORAD, a critical long non-coding RNA in human cancers. Life Sci..

